# Visualisation of microvascular flow in benign uterine disorders: a pilot study of a new diagnostic technique

**DOI:** 10.52054/FVVO.15.2.072

**Published:** 2023-06-30

**Authors:** M Frijlingh, R.A. de Leeuw, L.J.M. Juffermans, T van den Bosch, J.A.F. Huirne

**Affiliations:** Department of Obstetrics & Gynaecology, Amsterdam UMC, Vrije Universiteit Amsterdam, Amsterdam, the Netherlands; Amsterdam Reproduction and Development, Amsterdam, the Netherlands; Department of Obstetrics and Gynaecology, University Hospital Leuven, Leuven, Belgium; Department of Development and Regeneration, KU Leuven, Leuven, Belgium

**Keywords:** Ultrasound, microvascular flow, vascular architecture, uterine disorders, fibroids, endometriosis

## Abstract

**Background:**

Uterine disorders have clear overlapping symptoms and ultrasound discrimination is not always easy. Accurately measuring vascularity is of diagnostic and prognostic value. Power Doppler is limited to imaging only the larger vessels. Assessment of the microvasculature requires advanced machine settings.

**Objectives:**

In this pilot study, we aimed to test the feasibility of microvascular flow imaging of benign uterine disorders.

**Material and Methods:**

Two experienced gynaecologists (JH, RL) randomly applied power Doppler and MV- flowTM mode during a single day, in ten patients each visiting the outpatient clinic. Images of eight patients were labelled with a diagnosis by the attending physicians and collected as coded data.

**Main outcome measures:**

Microvascular flow images of normal uterine architecture including the fallopian tube, and of benign disorders such as fibroids, adenomyosis, endometriosis and uterine niches were collected. For both Doppler techniques, qualitative descriptive evaluation of the vascular architecture and a quantitative vascular index of fibroids were provided. Finally, we evaluated the effect of the cardiac cycle.

**Results:**

All microvascular flow images showed more distinctive vascular structures than visible on power Doppler. Calculating a vascular index for fibroids on 2D MV-flow^TM^ images was easily performed on-site. During the cardiac cycle a higher vascular index (VI 75.2) is obtained in systole as compared with diastole (VI 44.0).

**Conclusion:**

Microvascular flow imaging allowed detailed visualisation of the uterine vascular architecture and is easy to use.

**What is new?:**

Microvascular flow imaging may be of added value for diagnosing uterine disorders, as well as for pre- and post-operative assessment of suited surgical techniques. Yet, validation with histology and clinical outcomes is required.

## Introduction

Uterine disorders have clear overlapping symptoms and ultrasound discrimination is not always easy. A correct diagnosis is mandatory for optimal disease management and treatment (Van den Bosch et al., 2015; Vitale et al., 2021). Benign gynaecological disorders can be treated medically, surgically or by other minimally invasive techniques. For example, an effective uterine sparing method to treat uterine fibroids surgically, is laparoscopic myomectomy. However, perioperative it may become clear that the uterus is also affected by adenomyosis, which may result in major blood loss during myomectomy or makes it more difficult to find the cleavage planes (Tanos et al., 2018). Identification of the location of, for example, endometrioses is crucial, since removal of bowel-related endometriosis requires extensive surgical skills (Zanelotti and Decherney, 2017). Therefore, it is of utmost importance to identify and evaluate uterine disorder(s) correctly by imaging (De Franciscis et al., 2020). Measuring the degree of vascularity in fibroids is of prognostic value for growth potential and may be related to symptom severity (Keizer et al., 2021; Nieuwenhuis et al., 2018b). Specifically, assessment of the microvasculature may have important diagnostic and prognostic implications and may have a considerable role in predicting the most suited treatment, as well as follow-up after surgery.

In gynaecology, the most frequently used imaging technique is ultrasound (Loverro et al., 1999). Power Doppler allows for visualisation of lesion blood supply, for example, typical circumferential flow in fibroids and translesional vessels in case of adenomyosis (Harmsen et al., 2022; Van den Bosch et al., 2015). Yet, Doppler ultrasound has limitations. It enables the visualisation of mainly larger vessels and cannot detect the slow flow in micro vessels (Frijlingh et al., 2022). Measuring microvascular slow flow requires a higher frame rate and advanced filtering modes, as has been implemented in a number of high-end ultrasound machines. Ultrasound modalities to measure slow blood flow are at the discretion of ultrasound manufacturers: e.g., Superb Microvascular Imaging by Toshiba or Canon Medical Systems (Tokyo, Tokyo Metropolis, Japan), SlowflowHD by GE Healthcare (Zipf, Upper Austria, Austria) or MV- flowTM by Samsung Medison (Samsung Medison Co., Ltd., Seoul, South Korea).

The feasibility of imaging microvascular flow measured by MV-flow TM has recently been demonstrated in obstetrics, but its use in gynaecology has not been published yet (Chen et al., 2021; Dall’Asta et al., 2021; Malho et al., 2021). We aimed to visualise microvascular flow in benign uterine disorders, such as fibroids, adenomyosis, and endometriosis, as well as in the pre-operative assessment before niche repair. Qualitative descriptive evaluation of the vascular architecture for microvascular flow images, conventional B-mode and power Doppler images will be provided, alongside a quantitative vascular index for microvascular flow imaging and 3D power Doppler images. Finally, we will evaluate the effect of the cardiac cycle.

## Materials and Methods

We performed a pilot study using ultrasound data scanned at our outpatient clinic in the Amsterdam UMC, the Netherlands. The study was approved by the ethics committee of the Amsterdam UMC (Ref. No. W21_583 # 22.027). All ultrasound scans were performed by two experienced gynaecologists (JH, RL) with more than 10 years’ experience in advanced ultrasound evaluation of gynaecological disorders. They randomly applied power Doppler and MV-flow^TM^ mode during their consultation on September 6th, 2021. Most representable ultrasound images of benign uterine disorders were labelled with a diagnosis by the attending gynaecologist and collected as coded data by the researcher (MF). Decoding was only possible by the clinical team (JH, RL, MF). The coded data and informed consent were covered by the Status-study, a cohort study for the collection of medical and ultrasound data (Ref. No. W20_274).

Ultrasound scanning was performed, both in power Doppler (PD) and MV-flow^TM^ mode, using a HERA W10 system with a 3D EV2-10A or EV3- 10B endovaginal probe (2 - 10 MHz) (Samsung Medison Co., Ltd., Seoul, South Korea). The settings of the HERA W10 ultrasound included a 2D frequency of 2.0 – 8.2 MHz, gain 50 dB, pulse repetition frequency of 0.58 Hz, sensitivity of 10, filter 3, 3D quality ‘high2’, angle 90◦, Power maximal (Samsung Medison Co., Ltd., Seoul, South Korea).

**Figure 1:**
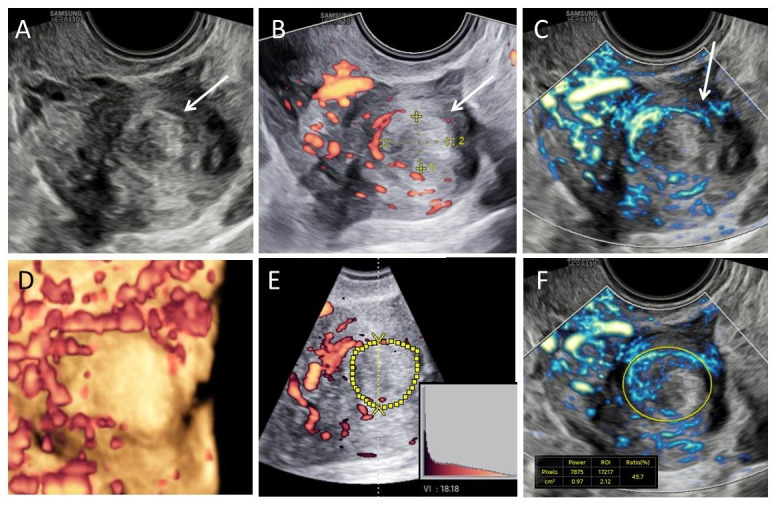
Representative images of a fibroid. A. Fibroid in B-mode; B. Fibroid in power Doppler mode; C. Fibroid in MV-flow^TM^ mode. Arrow indicates circumferential flow; D. 3D power Doppler reconstruction fibroid; E. How to determine the vascular index in 3D power Doppler mode, yellow circle depicts the fibroid as region-of-interest; F. How to determine the vascular index in MV-flow^TM^ mode.

As part of our routine, we started with uterine evaluation using 2D B-mode and vascular architecture was evaluated using PD-mode, both according to the Morphological Uterus Sonographic Assessment (MUSA) criteria (Van den Bosch et al., 2015). Next, MV-flow^TM^ mode was enabled and the gain was adjusted in case of artefacts or excessive delay. Vascular index (VI) obtained using both 3D power Doppler and MV-flow^TM^ mode. The VI represents the percentage of coloured pixels divided by all pixels (coloured and grey pixels). The VI was obtained using the manual selection of the region of interest using 3D PD according to standardised steps ((Frijlingh et al., 2022) (Figure 1D and 1E). This was performed offline using VOCAL (Virtual Organ Computer-aided AnaLysis) software Sonoview Pro- 1.6.2 (Samsung Medison Co., Ltd., Seoul, South Korea) according the previously described method (Nieuwenhuis et al., 2018a). The MV-flow^TM^ VI was calculated on site in real time on a 2D image (Figure 1F). Finally, we evaluated the effect of the cardiac cycle. Since this was only a feasibility and pilot study, we did not perform any statistical analysis.

## Results

### Patients

In September 2021 the two experienced gynaecologists (JH, RL) randomly applied power Doppler (PD) and MV-flow^TM^ mode during a single day, in ten patients each, who were visiting the outpatient clinic of the Amsterdam UMC, location AMC. These 20 patients were diagnosed with fibroids (≈50%), adenomyosis (≈30%), endometriosis (≈8%), niche (≈33%) and/or other disorders (≈4%). MV-flow images were successfully obtained in all cases. Eight patients were selected to illustrate visualisation of microvascular flow using MV-flow^TM^.

### Normal uterine vascular architecture

**Figure 2:**
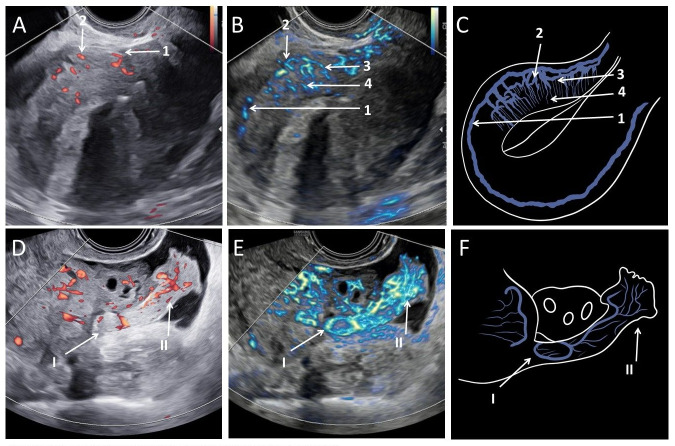
Representative images of vascular architecture of uterus and of a fallopian tube. A. Normal architecture measured by power Doppler mode with an arcuate (1) and radial (2) artery visible; B. Normal architecture visible by MV-flow^TM^ mode with an arcuate (1), radial (2), basal (3) and spiral (4) arteries clearly shown; C. Schematic overview normal uterine vascular architecture; D. Fallopian tube in power Doppler mode; E. Fallopian tube in MV-flow^TM^ mode; F. Schematic overview Fallopian tube. Arrows indicate the ampulla (I) and fimbriae (II) of the Fallopian tube.

A uterus is vascularised by the uterine and the ovarian arteries. Microvascular flow (MV-flow) images accurately display the uterine and ovarian arteries that form an anastomosis resulting in an arterial arcade located between the middle and outer myometrium (Cicinelli et al., 2004). The uterine vascularisation continues into the myometrium as radial arteries and in the endometrium as small spiral arteries (Hickey and Fraser, 2003), see Figure 2A-C.

MV-flow mode can also be used to image a fallopian tube (Figure 2D-F), identifying vessels continuing from the fallopian tube wall to the fimbriae. Power Doppler (PD) is only partially able to detect this flow.

### Vascular architecture in benign uterine disorders

#### Fibroids

Fibroids typically show circumferential flow and are circumscribed by a pseudo capsule of compressed tissue of the adjacent myometrium, whereas the centre of the fibroid is generally less vascularised (Van den Bosch et al., 2015). The typical circumferential flow with apparent avascular centre is clearly seen in both Doppler modes (Figure 1A-C). MV-flow imaging provides a more detailed impression of the vascular architecture compared to PD-mode, showing continuously visible vascular structures from the vascular arcade to the circumferential rim. Flow was detected in the entire vascular capsule surrounding the fibroid and displayed multiple, small branching vessels.

In order to quantify the observed vascular flow, a VI can be measured for both MV-flow imaging and PD-mode. A VI is generally higher in MV-flow^TM^ mode, than in PD-mode, particularly if focussed on the fibroid centre (Figure 1D-F).

#### Adenomyosis

**Figure 3:**
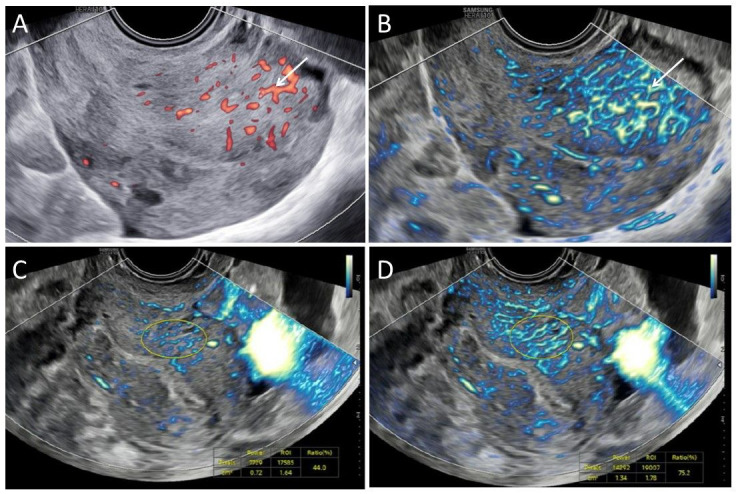
Representative images of a uterus with adenomyosis and influence of cardiac cycle. A. Adenomyosis in power Doppler mode; B. Adenomyosis in MV-flow^TM^ mode. Arrow indicates translesional flow in adenomyotic lesion; C. Adenomyosis visible in diastole, two dimensional (2D) microvascular flow vascular index 44,0; D. Adenomyosis visible in systole, 2D microvascular flow vascular index 75,2.

Ectopic endometrial tissue within the myometrium is called adenomyosis. Besides B-mode ultrasound criteria, translesional flow is considered to be a typical indirect sign of adenomyosis (Harmsen et al., 2022; Van den Bosch et al., 2015). Adenomyosis in PD-mode only reveals several larger vessels, whereas MV-flow imaging distinguished the hyper-vascularised region of adenomyosis from the adjacent myometrium, showing a typical irregular branching pattern, perpendicular to the serosa (Figure 3).

#### Endometriosis

**Figure 4:**
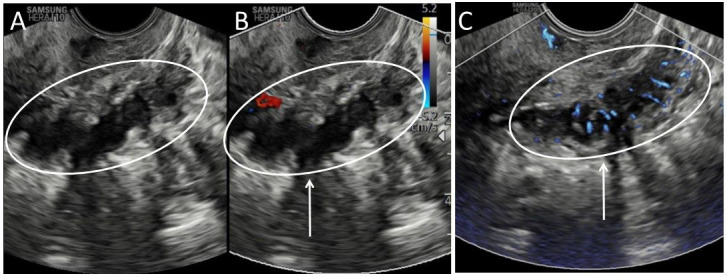
Representative image of a deep infiltrating endometriosis lesion. A. Endometriosis in B-mode; B. Endometriosis in Directional power Doppler mode; C. Endometriosis in MV-flow^TM^ mode. Arrows indicate deep endometriosis lesion with infiltrating vessels, only visible in MV-flow^TM^ mode.

Endometriosis is characterised by the presence of ectopic endometrial tissue outside the uterus (Van den Bosch et al., 2015). Endometrial tissue can be well-vascularised stroma but may also develop into less vascularised fibrotic tissue. In directional Doppler mode, no flow is displayed in the deep endometriosis lesion (Figure 4B). In MV-flow^TM^ mode, the blood supply of the deep endometriosis lesion is visible as equally spreading small vessels perpendicular to the bowel wall, infiltrating into the lesion and the submucosal layer of the bowel (Figure 4C).

#### Pre- and post-operative assessment of scar tissue

Scar tissue is characterised by fibrosis and the absence of blood flow. Scar tissue may develop after surgery such as a caesarean section or myomectomy. In the majority of patients, a niche in the uterine caesarean section scar can be visualised using saline or gel instillation. The use of Doppler may help to differentiate between a uterine niche, Nabothian cysts and adenomyosis, which has consequences for further management (Jordans et al., 2019). If a niche resection is considered, it is important to excise all fibrotic tissue to allow proper wound healing after surgery (Huirne et al., 2017). MV-flow imaging may be useful for preoperative estimation of the extent of fibrotic tissue.

**Figure 5:**
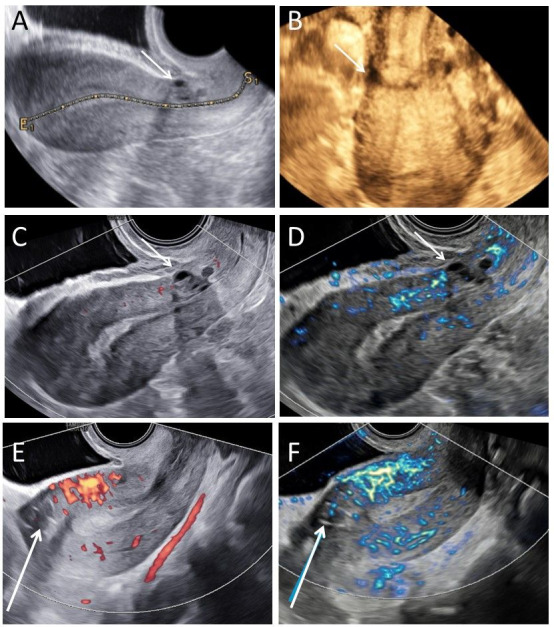
Representative images of pre- and postoperative assessment of scar tissue. A. Representative images of a complex niche shown in B-mode, sagittal view; B. Complex niche shown in three-Dimensional, coronal view; C. Complex niche shown in power Doppler mode; D. Complex niche shown in MV-flow^TM^ mode. Arrow indicates location of the complex niche and scarring tissue without vascularisation. E. Power Doppler image of the uterus 6 weeks after a laparoscopic myomectomy; F. Microvascular flow imaging after laparoscopic myomectomy, showing a hypovascularised area at the site of the incision, and clear flow at the proximal site. Arrow indicates stitches.

A complex niche is visible in Figure 5A-D with minimal PD-signal visible. MV-flow imaging clearly differentiates between less vascularised fibrotic tissue and well vascularised myometrium. Using microvascular flow imaging in a patient seen at six weeks after laparoscopic myomectomy of an eight-centimetre FIGO type 2-5 fibroid, we could identify a hypovascular area at the site of the incision and previous location of the sutures indicating local fibrosis (Figure 5E and 5F).

#### Cardiac cycle

During the cardiac cycle more flow can be observed during systole on all MV-flow recordings. Figure 3 shows an image of the uterus where the VI increases from 44.0 during diastole to 75.2 in systole. This is different from the minor variation observed during the cardiac cycle on the VI using 3DPD. Creating a 3D volume takes up to 10 seconds and includes multiple cardiac cycles, the VI thus represents a mean value between systole and diastole images.

## Discussion

MV-flow imaging may prove to be the missing tool for better recognition and distinction of gynaecological disorders with overlapping symptoms. MV-flow imaging is an easy-to-use Doppler technique allowing detailed visualisation of the uterine vascular architecture. It allows both the depiction of normal myometrial vascularity, as well as identifying abnormal vascularity in specific uterine disorders.

For imaging and diagnosing uterine fibroids MV-flow imaging may be useful in early detection, prediction of growth potential, selecting the appropriate treatment dependent on expected fibroid growth, as well as monitoring the results of minimally invasive treatment. Adenomyosis on the other hand, is often difficult to recognise using B-mode ultrasound and to discriminate from a fibroid. Adenomyotic lesions are characterised by translesional blood flow comprised of small vessels. MV-flow imaging is able to depict this typical vascular architecture and identify adenomyotic lesions. The same holds for endometriosis, especially small endometriosis spots or deep infiltrating endometriosis which can go undetected on B-mode and PD-imaging. Identification of the location of bowel-related endometriosis is important as it requires specialised operative care.

Although designed to depict MV-flow, this tool also allows identification of hypovascularised regions, in cases such as uterine scar after a caesarean section or after myometrial surgery. The use of MV-flow imaging technique may allow determination of the extent of fibrosis. During laparoscopic niche resections it is important to resect all fibrotic tissue to facilitate proper wound healing.

In contrast to hypovascular fibrotic areas, a fallopian tube is characterised by hyper-vascularisation. Imaging this feature may be helpful to differentiate between ovarian cysts and a hydrosalpinx.

Quantification of vascular flow via on-site calculation of the VI is quickly done with a single press of a button. As expected, a 2D MV-flow VI provides a higher VI than a 3DPD VI. However further studies are needed to define normal VI ranges using MV-flow imaging and PD-mode. MV-flow imaging VI is strongly affected by the cardiac cycle, whereas 3DPD VI is not. Influence of the cardiac cycle using 3DPD is just seen in case of a short scanning time and high vascularisation (Nieuwenhuis et al., 2018a). For standardisation we propose to use the systolic image using MV–flow imaging. Future studies are needed to assess relevant cut-off values for different disorders.

Recently, we described the importance of optimising settings such as gain, pulse repetition frequency and wall motion filter for PD-mode. Known standard settings for MV-flow imaging are low pulse repetition frequency, high frame rate and advanced wall motion filter. The optimal machine settings to evaluate the uterus using MV-flow imaging need to be defined.

Common Doppler artefacts are also applicable to MV-flow imaging, such as penetration issues. Neither PD-mode nor MV-flow^TM^ mode were able to display vascular flow signal in the posterior wall, causing the anterior wall to be seemingly more affected by adenomyosis (Figure 3B). Noise artefacts (Figure 4C) might be corrected by decreasing the gain and movement artefacts by increasing the tissue suppression (Figure 3C and 3D). From our limited experience, the recognised artefacts-errors were easily corrected.

Added clinical value of MV-flow imaging was demonstrated in two studies using ‘Superb Microvascular Imaging’ (Canon or Toshiba) in uterine fibroids treated with radiofrequency ablation or uterine artery embolisation (Coutinho et al., 2021; Samanci et al., 2021). In a series of 28 women, Superb Microvascular Imaging resulted in excellent interobserver agreement and was more sensitive than PD-ultrasound in predicting treatment response to uterine artery embolization. To date, no studies on microvascular imaging in gynaecology using MV-flow^TM^ (Samsung) or Slowflow (GE Healthcare) are available. MV- flowTM was investigated in four obstetric studies in high- and slow-flow placental vascularity (Chen et al., 2021), vascular high flow anomalies of the foetal heart (Malho et al., 2021), slow flow torcular herophili (Dall’Asta et al., 2021) and high-flow small vessels of the foetal head (Giuffrida et al., 2021). Although promising, the additional value and diagnostic accuracy of MV-flow, together with inter- and intra-observer agreement require future studies. One study evaluating inter- and intra- observer agreement between conventional Doppler and MV-flow imaging in the assessment of uterine fibroids is currently registered (ClinicalTrials.gov; reference number NCT05643339).

In our pilot study, we only included benign lesions. It would be interesting to study the added value of microvascular flow imaging to discriminate between benign and malignant lesions such as leiomyosarcomas. Current literature in non-gynaecological cancers is contradictory. In malignant hepatocellular carcinoma, MV-flow^TM^ mode was more sensitive and more accurate than colour or power Doppler in the detection of tumour vascularity (Kang et al., 2019). On the contrary, in thyroid nodules, no added value in the differentiation of benign versus malignant lesions was observed using MV-flow^TM^ (Yoo et al., 2020).

A limitation of our study is that microvascular flow was studied in non-systematically selected cases and interpreted by two experienced gynaecologists, thus inducing an inclusion bias. A second limitation is that we did not validate the depicted vascularity with histology. Future studies are needed to validate MV- flow imaging with histology and clinical outcomes, and the added value should be established before implementation in daily practice.

In conclusion, microvascular flow imaging is easy to use in gynaecology, provides clear images of uterine microvascular architecture of different uterine pathologies with a vascular index quickly acquired on-site. This ultrasound technique is promising, both as a diagnostic tool or as pre-operative assessment. However, validation studies and assessment of inter and intra- observer agreement should be performed before implementation in daily practice.

## References

[1] Chen X, Wei X, Zhao S (2021). Characterization of Placental Microvascular Architecture by MV-Flow Imaging in Normal and Fetal Growth-Restricted Pregnancies.. J Ultrasound Med.

[2] Cicinelli E, Einer-Jensen N, Galantino P (2004). The vascular cast of the human uterus: from anatomy to physiology.. Ann N Y Acad Sci.

[3] Coutinho C, Werner H, Lopes FP (2021). Cutting-edge application of ultrasound elastography and superb microvascular imaging in radiofrequency ablation of uterine fibroids.. J Ultrason.

[4] Dall’Asta A, Grisolia G, Volpe N (2021). Prenatal visualisation of the torcular herophili by means of a Doppler technology highly sensitive for low-velocity flow in the expert assessment of the posterior fossa: a prospective study.. BJOG.

[5] De Franciscis P, Riemma G, A Schiattarella (2020). Impact of Hysteroscopic Metroplasty on Reproductive Outcomes of Women with a Dysmorphic Uterus and Recurrent Miscarriages: A Systematic Review and Meta-Analysis.. J Gynecol Obstet Hum Reprod.

[6] Frijlingh M, Juffermans L, de Leeuw R (2022). How to use power Doppler ultrasound in transvaginal assessment of uterine fibroids.. Ultrasound Obstet Gynecol.

[7] Giuffrida A, Peixoto AB, Araujo Júnior E (2021). MV-Flow and LumiFlow: new Doppler tools for evaluating the microvasculature of the fetal head.. Radiol Bras.

[8] Harmsen MJ, Trommelen LM, de Leeuw RA (2022). Multidisciplinary view on uterine junctional zone in uteri affected by adenomyosis: explaining discrepancies between MRI and transvaginal ultrasound images on a microscopic level.. Ultrasound Obstet Gynecol.

[9] Hickey M, Fraser I (2003). Human uterine vascular structures in normal and diseased states.. Microsc Res Tech.

[10] Huirne JAF, Vervoort AJMW, Leeuw R (2017). Technical aspects of the laparoscopic niche resection, a step-by-step tutorial.. European journal of obstetrics, gynecology, and reproductive biology.

[11] Jordans IPM, de Leeuw RL, Stegwee SI (2019). Niche definition and guidance for detailed niche evaluation.. Acta Obstet Gynecol Scand.

[12] Kang HJ, Lee JM, Jeon SK (2019). Microvascular Flow Imaging of Residual or Recurrent Hepatocellular Carcinoma after Transarterial Chemoembolization: Comparison with Color/Power Doppler Imaging.. Korean J Radiol.

[13] Keizer AL, Niewenhuis LL, Hehenkamp WJK (2021). Fibroid vascularisation assessed with 3D Power Doppler as predictor for fibroid related symptoms and quality of life; a pilot study.. Facts Views Vis Obgyn.

[14] Loverro G, Bettocchi S, Cormio G (1999). Transvaginal sonography and hysteroscopy in postmenopausal uterine bleeding.. Maturitas.

[15] Malho AS, Ximenes R, Ferri A (2021). MV-Flow and LumiFlow: new Doppler tools for the visualization of fetal blood vessels.. Radiol Bras.

[16] Nieuwenhuis LL, Hehenkamp WJK, Brolmann HAM (2018). 3D power Doppler in uterine fibroids; influence of gain, cardiac cycle and off-line measurement techniques.. J Obstet Gynaecol.

[17] Nieuwenhuis LL, Keizer AL, Stoelinga B (2018). Fibroid vascularisation assessed with three-dimensional power Doppler ultrasound is a predictor for uterine fibroid growth: a prospective cohort study.. BJOG.

[18] Samanci C, Ozkose B, Ustabasioglu FE (2021). The Diagnostic Value of Superb Microvascular Imaging in Prediction of Uterine Artery Embolization Treatment Response in Uterine Leiomyomas.. J Ultrasound Med.

[19] Tanos V, Berry KE, Frist M (2018). Prevention and Management of Complications in Laparoscopic Myomectomy.. Biomed Res Int.

[20] Van den Bosch T, Dueholm M, Leone FPG (2015). Terms, definitions and measurements to describe sonographic features of myometrium and uterine masses: a consensus opinion from the Morphological Uterus Sonographic Assessment (MUSA) group.. Ultrasound Obstet Gynecol.

[21] Vitale SG, Laganà AS, Caruso S (2021). Comparison of three biopsy forceps for hysteroscopic endometrial biopsy in postmenopausal patients (HYGREB-1): A multicenter, single-blind randomized clinical trial.. Int J Gynaecol Obstet.

[22] Yoo J, Je BK, Choo JY (2020). Ultrasonographic Demonstration of the Tissue Microvasculature in Children: Microvascular Ultrasonography Versus Conventional Color Doppler Ultrasonography.. Korean J Radiol.

[23] Zanelotti A, Decherney AH (2017). Surgery and Endometriosis.. Clin Obstet Gynecol.

